# Linezolid Adsorption on Filters during Continuous Renal Replacement Therapy: An In Vitro Study

**DOI:** 10.3390/ph17101317

**Published:** 2024-10-02

**Authors:** Krzysztof Nosek, Milena Samiec, Hubert Ziółkowski, Paulina Markowska-Buńka, Mirosław Czuczwar, Michał Borys, Dariusz Onichimowski

**Affiliations:** 1 Department of Pharmacology and Toxicology, Faculty of Medicine, University of Warmia and Mazury, Al. Warszawska 30, 10-082 Olsztyn, Poland; 2 Department of Anesthesiology and Intensive Therapy, Faculty of Medicine, University of Warmia and Mazury, Al. Warszawska 30, 11-082 Olsztyn, Poland; 3 Department of Pharmacology and Toxicology, Faculty of Veterinary Medicine, University of Warmia and Mazury, Oczapowskiego 13, 10-718 Olsztyn, Poland; 4 2nd Department of Anaesthesiology and Intensive Therapy, Medical University of Lublin, 20-059 Lublin, Poland

**Keywords:** adsorption, linezolid, continuous renal replacement therapy, dialysis filter

## Abstract

**Background:** Renal replacement therapy (RRT), widely used in the treatment of renal injury during sepsis, aims to eliminate the toxins and proinflammatory cytokines involved in the pathomechanism underlying septic shock. Dialysis filters are characterized by a high adsorption potential for cytokines in RRT in the case of septic renal injury. For the treatment of sepsis with antibiotics, it is of key importance to achieve the desired values of PK/PD indices. Continuous renal replacement therapy (CRRT) may affect antimicrobial clearance, increasing their elimination in some cases. **Methods:** The aim of this study was to determine the degree of adsorption for linezolid on three different types of filters used in CRRT. In our in vitro study, a continuous veno-venous hemofiltration (CVVH) was conducted using three types of filters: polysulfone (PS), polyethyleneimine-treated polyacrylonitrile (PAN PEI), and non-PEI-treated polyacrylonitrile (PAN). Each type of filter was used in three CVVH cycles, involving the use of 600 mg of linezolid dissolved in 700 mL of bovine blood or in 700 mL of 0.9% NaCl. In each case, the total volume of the obtained solution was 1000 mL. Blood samples were collected at particular time points to measure their drug concentration. The differences in mean drug/NaCl adsorption and drug/blood adsorption were determined using a one-way ANOVA with multiple comparisons via Tukey’s post hoc test; a *p*-value of <0.05 was considered significant. **Results**: A significant adsorption of linezolid was found for PAN PEI filters, both in samples obtained from bovine blood and 0.9% NaCl solutions, at the endpoint. In PAN PEI samples, the concentration of linezolid in 0.9% NaCl solutions decreased from 594.74 μg/mL to 310.66 μg/mL after 120 min (the difference was established at 52%). In blood samples, the initial concentration was 495.18 μg/mL, which then decreased to 359.84 μg/mL (73% of the beginning value). No significant adsorption was demonstrated on PAN or PS filters. **Conclusion:** There is a need for in vivo research to confirm the effect of filter type on linezolid concentration in patients undergoing CRRT.

## 1. Introduction

According to SSC 2021 guidelines, the prognosis for patients with sepsis depends largely on the prompt institution of antibiotic therapy and adequate fluid intake. The appropriate selection of an antibiotic and its optimal dosage are of primary importance. It is, therefore, necessary to consider the pharmacokinetic and pharmacodynamic properties of a drug with a view to achieving optimal pharmacokinetic/pharmacodynamic (PK/PD) indices. Sepsis and septic shock are the most common causes of acute kidney injury (AKI) [1] and the pathomechanism of AKI involves hypoperfusion or compromised renal perfusion, inflammation, the release of proinflammatory cytokines, and the compromised function of renal cell mitochondria [1,2]. The implementation of continuous renal replacement therapy, apart from replacement or support for renal function in septic patients, enables the elimination of inflammatory mediators from the system, enhances the stability of the circulatory system, improves organ perfusion, and facilitates a gradual reduction in plasma osmolality to avoid DDS (dialysis disequilibrium syndrome) [2,3]. To increase toxin adsorption, polyethyleneimine-treated polyacrylonitrile (PAN PEI) filters, capable of adsorbing small-, medium-, and large-sized molecules, are introduced into RRT. It has been demonstrated that proinflammatory cytokines (tumor necrosis factor (TNF)-α; interleukins (IL)-1β, IL-6, IL-8, and IL-10) also undergo adsorption on this type of filter. Moreover, a negatively charged polyacrylonitrile (PAN) membrane makes it possible to bind positively charged molecules, including drugs. PAN filters treated with polyethyleneimine (PEI) generate weaker negative charges, reducing the production of bradykinin but still make it possible to bind negatively charged drugs, such as heparin [4,5]. With polysulfone (PS) filters, during CRRT, the elimination of molecules up to 30 kD is possible, while the capacity of these filters to eliminate cytokines is much smaller than with acrylonitrile filters [4]. Currently, the abovementioned are the most widely utilized dedicated CRRT filters, both in our country and across Europe [6]. Linezolid is an antibiotic with a molecular weight of 337.35 Da and with normal pH; it is an electrically neutral molecule [7]. The efficacy of linezolid therapy depends on how long the concentration remains above the MIC (T > MIC), as well as on the ratio of the 24 h area under the concentration–time curve to MIC (AUC24 > MIC) [8,9]. Intensive therapy patients receiving standard linezolid doses (600 mg every 12 h) are reported to have inadequate plasma drug concentrations, which implies worse prognosis, so introducing increased drug doses into the therapy has been considered [10]. One of the causes which might dictate the need to change the dosing regimen of linezolid may be the adsorption of the drug on the filter in patients undergoing CRRT [11,12,13]. In our study, to eliminate these doubts, we decided to assess the degree of linezolid adsorption on a range of filters: PS, PAN, and PAN PEI. To exclude the effect of plasma proteins on the degree of adsorption, we compared solutions of bovine blood and 0.9% NaCl containing 600 mg of linezolid.

## 2. Results

The analysis of the drug concentration in the control solutions did not show a decrease in concentration in any case, suggesting that antimicrobial studies did not undergo spontaneous degradation in the test solution used for CVVH. In all blood samples, the albumin values did not differ significantly from one another and equaled 410.11, 412.6, and 393.79.

The greatest and most significant adsorption was noted for the PAN PEI membrane (Table 1). In the samples with bovine blood, a significant difference was demonstrated on the PAN PEI filter over time between the starting value and each time point (*p* < 0.05). The value at 120 min (T120) constituted 73% of the initial value (Table 2).

**Figure 1:**
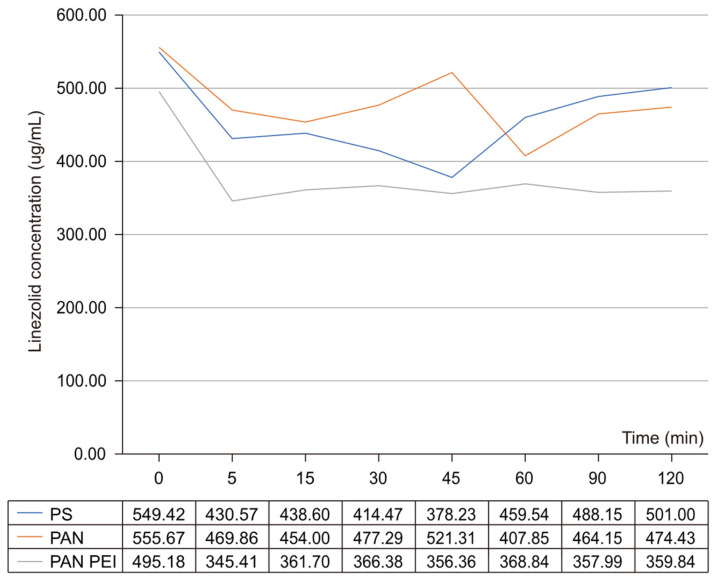
Concentration of linezolid in the blood during CVVH using different kinds of membranes: PS—polysulfone membrane; PAN PEI—polyacrylonitrile membrane with polyethylene imine; and PAN—polyacrylonitrile membrane.

The lowest drug concentration for the PAN membrane during CVVH with a blood solution was seen after 60 min of the experiment (Figure 1). The concentration values gradually increased in two subsequent samples while failing to reach the initial level: the difference between the starting concentration and the endpoint was 15% (Table 2).

Regarding the PAN PEI membrane, the drug concentration fell after 5 min of the test and remained at a relatively stable, albeit low level, until the end of the test. For the PS membrane, the concentration of linezolid in the blood solution was the lowest at 45 min before it increased gradually, and at the endpoint, it did not differ significantly from the starting value.

The level of the plasma protein binding for linezolid is approximately 30% [14], which is why CVVH was also conducted for the solution of linezolid in 0.9% NaCl. For solutions with 0.9% NaCl, the greatest and most significant adsorption was demonstrated for the PAN PEI membrane (Table 3).

**Figure 2:**
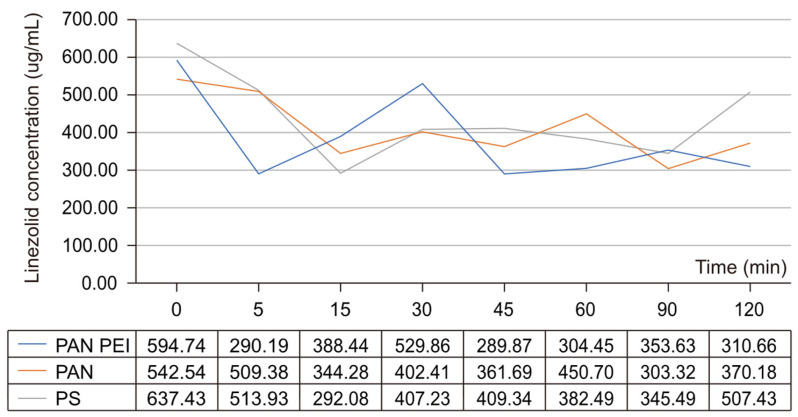
Concentration of linezolid in the solution of 0.9% NaCl during CVVH using different kinds of membranes: PS—polysulfone membrane; PAN PEI—polyacrylonitrile membrane with polyethylene imine; PAN—polyacrylonitrile membrane.

The lowest concentration of the antibiotic was seen at 5 and 45 min. At the endpoint at 120 min, the difference between the concentration at 0 and 120 min was 48%, and this was statistically significant (Figure 2) (Table 4).

For the PAN filter, only at 90 min was there a considerable fall in concentration (*p* < 0.05) to 303.32 μg/mL, which then increased at 120 min to 370.18 μg/mL , from an initial concentration of 542.54 μg/mL. For the PS filter, a significant decrease in concentration was demonstrated between 15 and 90 min, while the endpoint value was 507.43 μg/mL and was 20% lower than the initial value of 637.43 μg/mL (Table 5).

In the ANOVA + Tukey post hoc test, a statistically significant variability in the area under the curve (AUC) was demonstrated between PAN PEI vs. PS filters and PAN PEI vs. PAN filters in the blood samples (Table 4 and Table 6). The lowest AUC values were found on the PAN PEI filter in blood samples. The samples with 0.9% NaCl also showed the lowest AUC for PAN PEI filters, but for those samples, the values between the filters were closer than in the blood samples (Table 4 and Table 7).

The extraction ratio (Table 8) in our study was measured, and the results are shown below at 15% comparing samples in water (without matrix). Total recovery standards (Table 9) were also fulfilled with a standard variation below 15%.

## 3. Discussion

The pharmacokinetics and pharmacodynamics of many antibiotics may change in critically ill patients with multiorgan failure. Changes in antibiotic concentrations are particularly hazardous in septic patients, in whom drug concentrations between doses may fall below the MIC, or the AUC/MIC ratio may be too low [15]. This phenomenon is more pronounced when patients in septic shock develop renal failure and need renal replacement therapy. In patients who are hemodynamically unstable and undergoing septic shock, the preferred option remains to provide continuous renal replacement therapy (CRRT).

To obtain better therapy effects in patients requiring CRRT, the right dose of antibiotics and the regimen accounting for altered pharmacokinetics of the drug (PK) must be selected. However, existing studies on the changed antibiotic pharmacokinetics during CRRT are scarce [16]. The factors which affect the PK/PD of the drug during CRRT include preserved residual diuresis, drug distribution volume (the greater it is, the smaller the elimination of the drug during CRRT), extrarenal clearance, the level of drug binding to proteins, the duration and parameters of CRRT, blood flow rate, and types of filters [17]. The adsorption of the drug on the filter depends on the electrical charge of the membrane as well as the drug, the material of the membrane, its structure, and the degree of drug binding to plasma proteins [16,18].

Linezolid is an antibiotic with a small molecular weight (337 Da) and a neutral electrical charge. Its volume of distribution accounts for a total body water content of 40–50 L, and owing to its moderately lipophilic nature, it undergoes minimal changes during sepsis. Its plasma half-life ranges between 3.4 and 7.4 h. Approximately 30% of the drug binds to plasma proteins, while about 70% constitutes an unbound fraction [16,19,20].

The renal clearance of linezolid is approximately 30%. However, in patients with sepsis whose life is threatened, drug pharmacokinetics may change considerably, particularly in patients with AKI undergoing CRRT. The clearance of linezolid during CRRT differs depending on the kind of therapy, and for CVVH (the method used in our study), it was 1.2–3.2 L/h (the total drug clearance being 6.4–14.8 L/h) [17,19]. The efficacy of linezolid action depends on time (T > MIC) and the ratio of the 24 h area under the concentration–time curve during period to the MIC (AUC_0–24_/MIC) [19,21]

Our in vitro study was conducted on three types of filters commonly used in clinical practice: PS, PAN, and PAN PEI. We used bovine blood as a model as it was most closely imitated in vivo conditions. The albumin values for all the solutions were similar: 410.11 mg, 412.6 mg, and 393.79 mg, respectively. To exclude the possibilities of drug elimination other than its adsorption on the dialysis filter membrane, there were more tests carried out with the drug dissolved in a 0.9% NaCl solution, which was subjected to CVVH. In addition, a control test without CVVH was conducted to exclude spontaneous drug degradation. Significant adsorption was obtained for the PAN PEI filter, both in samples containing bovine blood plasma, as well as 0.9% NaCl. Taking into account the AUC values obtained in our study for blood samples, a statistically significant difference in values was found for PAN PEI filters.

A review article by Villa G et al. [19] mentions the surface area of the filter as a significant factor: the greater it is, the more drug clearance occurs. Carcerelo et al. [22] demonstrated that similar values of total extracorporeal drug clearance are obtained for the PAN filter and for the PS filter with a surface area almost twice the size as the filter with the PAN membrane. The aim of our study was to determine and compare drug adsorption regardless of the filter surface area but in relation to its type. This is why we used filters with the greatest available adsorption surface area, which were also similar in size. The review article from 2016 [19] points out that, in septic patients with AKI undergoing CRRT, only one publication found an optimal AUC/MIC ratio for pathogens with dedicated MIC > 4 mg/L. Regarding the PK/PD of linezolid in patients treated with CRRT, Villi G. et al. [19] found a decrease in the min. concentration, while the max. concentration was similar to or even higher than those mentioned in the literature. Bandin-Vilar et al. [23] reported clearance of linezolid of 30% in patients treated with RRT, with subtherapeutic drug concentrations more common than supratherapeutic values.

The publication by Sartori et al. [24] was based on the analysis of the linezolid adsorption on polysulfone (PS) filters. The results of this analysis proved that there was a decrease in drug concentration during the first 10 min, and then its concentration increased both in saline and blood samples—a rebound phenomenon thus occurred. A similar phenomenon was observed in our study involving the use of polysulfone membranes, although it was delayed (Figure 1 and Figure 2). We think that the increase in drug concentration results from the release of the antibiotic after the prior saturation of the filter. In a publication by Meyer et al. [25], PK parameters were comparable for patients undergoing CVVH involving the use of PS filters for healthy individuals.

In an article by Hiraiwa T et al. [12] a sharp fall in linezolid concentration was reported between 0 and 15 min on three types of filters, including AN69 ST (PAN PEI) and PS. The PEI on the PAN membrane in AN69ST filters changes the adsorption properties of the membrane, increasing the adhesion of some complement components and β2macroglobulines and also decreasing the negative charge of the membrane. The PAN PEI hydrogel structure allows adsorption not only on the surface of the membrane but also within it, which translates into its greater ability to remove cytokines [18,22,26]. The analysis of the results we obtained shows that the addition of PEI to the PAN membrane had a significant effect on the adsorption of linezolid, both in 0.9%NaCl and blood solutions.

It seems that using PAN PEI filters plays an important role here, as they are negatively charged; multiple sulfone groups attract water, forming a hydrogel structure, which is characterized by high diffusion permeability. The chemical composition of the AN69ST (PAN PEI) membrane enables the adsorption of proteins with a low molecular weight, and the high water content in hydrogel makes the polymer chains easily accessible [27]. The addition of PEI to PAN filters reduces the negative charge of the filter, which may translate into a decreased the adsorption of drugs with a positive charge [5].

The publication by Matthew S. Dryden reports the administration of linezolid at the dose of 600 mg every 12 h, resulting in linezolid concentrations >MIC90 for sensitive pathogens maintained until a subsequent dose is administered, with the threshold values observed between 10 and 12 h for *Staphylococcus* infection [15]. There are several factors which may affect the PK/PD ratio for linezolid, leading to subtherapeutic drug concentrations. These risk factors include CRRT and adsorption of antibiotics on a filter. There is a need for further research to reach a consensus on modifying linezolid therapy in CRRT patients to minimize the risk of obtaining subtherapeutic doses. Even short periods of subtherapeutic concentrations of a time-dependent antibiotic, such as linezolid, may lead to therapeutic failure, particularly for less sensitive pathogens, and contribute to growing bacterial resistance. Developing new recommendations requires further research in the clinical setting. A. Corona et al. emphasized that with the need to adjust antibiotic dosage in patients undergoing CRRT, the category for a time-dependent vs. concentration-dependent antibiotic must be considered as the benefits will be derived from modifying the treatment strategy. For a time-dependent antibiotic, a continuous infusion or prolonged administration of the drug will prove more beneficial, while for concentration-dependent antibiotics, a single dose of the drug should be increased [17].

## 4. Materials and Methods

**Figure 3:**
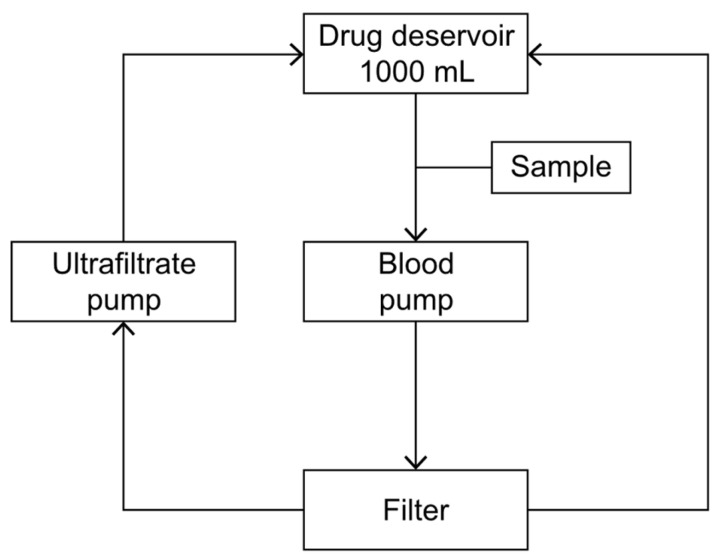
Diagram showing the CVVH circuit used in the in vitro study.

The aim of this study was to assess the degree of linezolid adsorption for three types of filters: polysulfone PS AV1000S (Fresenius Medical Care, St. Wendel, Germany), the polyethyleneimine-treated polyacrylonitrile filter AN69ST150 (Baxter International Inc., Deerfield, IL, USA), and non-eneimine polyacrylonitrile filter AN69HF M150 (Baxter International Inc., USA) using a continuous veno-venous hemofiltration (CVVH) circuit filled with blood or 0.9% NaCl. The test was carried out on a device used for CRRT in a clinical setting (Multifiltrate, Fresenius Medical Care, Germany) with a filtration kit (MultiFiltrate Kit 7 HV-CVVH 1000 Fresenius). Filters PS AV1000S, AN69ST150, and AN69HF M150 with the surface areas of 1.8 m^2^, 1.5 m^2^, and 1.5 m^2^, respectively, were inserted, as presented in Figure 3.

The total volume of the blood compartment in the kit and filter equaled approximately 200 mL. The parameters of the CVVH procedure were set at a blood flow rate of 100 mL/min and an ultrafiltration rate of 600 mL/h. Prior to the commencement of this study, the circuit was filled with 0.9% NaCl without an antibiotic. Subsequently, a reservoir was connected, containing bovine blood or 0.9% NaCl solution with an antibiotic. To prevent the thrombosis of fresh bovine blood, 30 mL of 7.4% sodium citrate was added to each liter of blood immediately after it was obtained. The amount of linezolid used in the test was 600 mg, which is the standard single dose used in clinical practice. The drug was dissolved in a reservoir filled with bovine blood or 0.9% NaCl, respectively, to obtain a total volume of 1000 mL. After CVVH was started, the first 200 mL of fluid (0.9% NaCl solution) was removed. During the test, the ultrafiltration fluid was continuously returned to the reservoir containing blood or 0.9% NaCl with the antibiotic. To maintain the temperature of recirculating fluid in the return line within the range of 35 to 37 °C, a GUARDIAN 5000 OHAUS hotplate stirrer was used. For every filter type and solvent, three study cycles were run. Both from 0.9% NaCl and bovine blood solutions undergoing CVVH, 3 mL solution samples were collected from the pump port of the CVVH circuit at 0, 5, 15, 30, 45, 60, 90, and 120 min, starting with the moment of preparing the solutions. Two control tests were carried out with the aim of assessing the spontaneous degradation of the drug. Solutions of 1000 mL total volume each were prepared, containing 600 mg of linezolid with bovine blood or 0.9% NaCl as solvents, respectively. The solutions were then heated to 35–37 °C and, maintaining a stable temperature, were left without conducting CVVH. This was followed by the collection of samples for analysis at the time points indicated above. The samples obtained from the bovine blood solution were centrifuged at 3000 rotations/min immediately after collection for the purpose of plasma separation. The obtained plasma was then frozen at −80 °C and, still frozen, delivered to a laboratory to determine the antibiotic concentration.

### 4.1. Drug Analysis

#### 4.1.1. Chemicals and Reagents

Analytical standards for LIN (linezolid) and deuterium-labeled LIN-d3 (for use as an internal standard, IS) were purchased from Sigma-Aldrich (Darmstadt, Germany) and Toronto Research Chemicals (Toronto, ON, Canada), respectively. Formic acid, acetonitrile, and water, all of mass spectrometry grade, were purchased from Sigma-Aldrich (St. Louis, MO, USA). Stock solutions (2 mg/mL for LIN and 1 mg/mL for IS) were prepared by dissolving the LIN and IS in methanol. Working solutions of the drugs for calibration curves (0.5 µg/mL, 2.5 µg/mL, 5 µg/mL, 25 µg/mL, 50 µg/mL, 100 µg/mL, 250 µg/mL, 500 µg/mL for LIN, and 50 µg/mL for IS) were prepared by diluting the standard solutions in methanol.

#### 4.1.2. Chromatography

The analysis was performed with Acquity UPLC System I-Class Plus coupled with a Xevo TQ-XS tandem mass spectrometer (MS/MS) (Waters, Milford, MA, USA). The chromatographic separation of both analytes was performed on an Acquity UPLC HSS T3 column (Waters, Milford, USA) (100 × 2.1 mm) with a particle size of 1.8 µm that was maintained at 35 °C. The mobile phase consisted of 0.1% formic acid in water (phase A) and 0.1% formic acid in acetonitrile (phase B), and the pump was set for gradient elution as follows: 0–0.75 min—10% phase A; 0.75–2.25 min—linear gradient to 100% phase A; and 2.25–3.00 min—linear gradient to 10% phase A. The duration of the entire analysis was 5.50 min, the injection volume was 1 µL, and the autosampler temperature was 15 °C. Detection was performed in the positive ion mode and MRM mode, and the transitions were set to 338.17 *m*/*z*→296.10 *m*/*z* for LIN and 341.17 *m*/*z*→297.10 *m*/*z* for IS. The basic mass spectrometry parameters were as follows: ionization mode—positive electrospray; desolvation gas—nitrogen; desolvation temperature 350 °C; desolvation gas flow—1000 L/h; source temperature 150 °C; collision gas—argon; collision energy (for both compounds)—17 eV; cone voltage—26 V; capillary voltage—0.5 kV; dwell—0.025 s; and delay—0.003 s.

For our experiments, we used two types of matrices: plasma and 0.9% NaCl. Both types of samples for LIN determination were prepared according to the method developed in our laboratory. As we were using mass spectrometry, we chose not to use a classical extraction procedure and used only a protein precipitation technique.

The 100 µL samples were thawed at room temperature (the samples for calibration curves were spiked with 10 µL of LIN) and mixed in a vortex mixer at 1000 rpm for 5 s. Next, 600 µL of acetonitrile (with 5 µg/mL of IS for calibration and 50 µg/mL of IS for experimental samples) was added for protein precipitation, and the samples were mixed at 3000 rpm for 10 s. After this, all samples were centrifuged at 20,000× *g* for 10 min at 4 °C.

At this point, to avoid the contamination of the LC-MS/MS (the concentrations of LIN in this experiment were more than 10 times higher than those achieved in clinical practice), the experimental samples (plasma and 0.9% NaCl) were subjected to an additional step of 10-fold dilution with water, unlike the calibration standards. This extra dilution was achieved by transferring 20 µL of the supernatant into a clean probe and diluting it with 180 µL of LC/MS water (the results from the analysis of the experimental samples were thus later multiplied by 10 to calculate the actual results). Finally, all samples (from the experiment and for calibration) were filtered through a 0.22 µm nylon syringe filter (13 mm in diameter) into chromatographic total recovery vials and injected into the chromatographic system.

The analytical method was validated according to Markowska et al. 2021 [28]. During the validation procedure, we used samples without any additional dilution procedure for the experimental samples that are described above (our experimental samples were diluted approximately 60 times, and we thus performed only the matrix effect tests for NaCl). We could thus measure the matrix effect precisely, and this method can also be used to analyze LIN in clinical practice. The following parameters were determined: linearity (an eight-point curve prepared and analyzed three times at one-day intervals), accuracy, precision (repeatability/intra-day precision and intermediate precision/inter-day precision, determined by preparing analyte concentrations at the three QC points and the LLOQ, which were all within the range of the standard curve; this was performed three times at one-day intervals in six replicates together with the IS), LLOQ, selectivity, recovery (six replicates of LLOQ and HQC points were prepared, in which the analytes were added to the plasma either before or after extraction), matrix effect (LIN and IS were added to the phase obtained following the extraction of an empty matrix in six replicates for LLOQ and HQC points and were compared with the signal of LIN and IS added to a mixture of water and ACN), carry-over (six replicates of HQC and six blank samples were prepared, and the blank sample was analyzed after each HQC sample analysis), and stability (without a long-term freeze–thaw stability test, but including freeze–thaw stability, autosampler stability, working standard and stock stability, and sample processing-temperature stability, always compared to freshly prepared samples; all these tests were performed by preparing analyte concentrations at the three QC points and the LLOQ, which were all within the range of the standard curve; this was performed three times, at specified time intervals, in six replicates together with IS). The acceptance criteria were based on Markowska et al. (2021) [28].

The lowest limit of quantitation (LLOQ) equaled 0.05 μg/mL ± 0.005 (the signal-to-noise ratio was no lower than 6:1). To prepare a calibration curve, plasma free from LIN was used, which was obtained from blood drawn from clinically healthy subjects. The curve included eight points at 0.05, 0.25, 0.50, 2.5, 5.0, 10.0, 25.0, and 50.0 μg/mL, of which three points served to demonstrate QC: low-quality control (LQC—0.25 μg/mL), medium-quality control (IQC—2.5 μg/mL), and high-quality control (HQC—50.0 μg/mL). The LIN calibration curve was linear. The coefficient of determination, r^2^, was above 0.99 for all the calibration curves. The differences between particular control points were 1.68–4.91% for accuracy, and the coefficient of variation for precision was within 1.75–7.23% for individual points. The specificity of the method was determined via an analysis of six samples of plasma free from LIN, which showed no significant peaks at the retention times of LIN and IS (2.20 min). In addition, there were no “ghost” peaks after the injection of six replicates of HQC (with IS) and six blank samples, which indicates that the chromatographic system did not carry over any analytes. With this method, the total recovery was 95% ± 8.9 for LIN and 97.1% ± 6.8 for IS. LIN was stable after 24 h in an autosampler at 15 °C (the increase/decrease in the concentration in the QC samples was ± 10.45–13.21%), after 3 h at the sample processing temperature (±2.38–9.26%), during the three cycles of thawing and freezing after 24, 48, and 480 h (±4.1–14.8%), and in the prepared stock and working standards stored in a refrigerator (4 °C) for 7 consecutive days (±1.14–14.12%). The matrix, which was plasma and 0.9% NaCl, did not demonstrate any significant effect on the signal from the detector, which was verified by analyzing the signal from the matrix with and without the LIN standard or by comparing the signal after adding the same concentration of LIN to water and to the matrix (the increase/decrease was ±7.35–14.52%).

### 4.2. Statistics

Descriptive statistics were performed. The normal distribution of continuous variables was tested using the Shapiro–Wilk test, while the differences in mean drug/NaCl adsorption and mean drug/blood adsorption between STL, ML, and PSL at different time points (0, 5, 15, 30, 45, 60, 90, 120) were determined using one-way ANOVA with multiple comparisons via Tukey’s post hoc test. The differences in mean drug/NaCl adsorption and drug/blood adsorption during the time for STL, ML, and PSL were measured using a repeated-measures ANOVA with multiple comparisons via Tukey’s post hoc test. A *p*-value < 0.05 was considered significant. The data analysis was conducted using Statistica (data analysis software), version 13 (http://www.statsoft.pl/staistica_13/ accessed on 1st January 2024)

## 5. Conclusions

This study covers a broad concept of testing the adsorption of antibiotics on filters during CRRT. In our studies so far, we have analyzed antibiotics from other groups, such as ciprofloxacin, gentamicin, vancomycin, and tigecycline [18], and we still plan to conduct analyses on antibiotics representing some other groups. Due to their pathophysiology, septic patients have a greater risk of achieving subtherapeutic antibiotic concentrations, and these differences may be clinically significant, particularly in patients undergoing CRRT, and depend, among other things, on the size of the antibiotic molecule and its charge. The institution of CRRT may cause changes in antibiotic pharmacokinetics through various mechanisms, including drug adsorption on a filter. In our study, the adsorption of linezolid on three types of membranes (PS, PAN, and PAN PEI) was compared. The adsorption of linezolid on the PAN PEI filter was found to be significant, and it may be of importance in the clinical setting. Data from both our study and the literature led us to conclude that an increased linezolid dosage should be considered, e.g., by introducing shorter intervals between the doses once CRRT is started or alternatively by doubling the dose of the antibiotic as CRRT is commenced or when the filter is replaced. In vivo studies are, therefore, mandatory to resolve this issue.

## 6. Limitations

The duration of the test was shorter than the recommended interval between linezolid doses (12 h), which failed to correspond to the changes in the PK/PD of linezolid. Further research should be conducted involving drug dosage in the clinical setting and the frequency of dialysis filter changes needed.

## Figures and Tables

**Table 1:** ANOVA for repetitive measurements of blood samples.

	PAN PEI		PAN		PS	
*p*	0.0002	Significant differences between zero and every one measurement	0.07	Significant differences between 0 and 60	0.06	Significant differences between 0 and 45

**Table 2:** Difference between final and initial linezolid concentrations in beef blood samples—percentage value.

Membrane Type	Concentration T0	Concentration T120	Difference T120/T0 (%)
PS	549.42	501.00	91%
PAN	555.67	474.43	85%
PAN PEI	495.18	359.84	73%

**Table 3:** ANOVA for repetitive measurements in 0.9% NaCl samples.

	PAN PEI		PAN		PS	
*p*	<0.001	Significant differences: 0 vs. 5, 15, 45, 60, 90, 120	0.01	Significant differences: 0 vs. 90 (*p* = 0.02)	0.002	Significant differences: 0 vs. 15, 30, 45, 60, 90

**Table 4:** AUC values on different types of filters in blood and 0.9% NaCl samples.

AUC(0-t) Values in Blood Samples			AUC(0-t) Values in 0.9% NaCl Samples		
Membrane Type	Mean (mg·min/L)	SD	Membrane Type	Mean (mg·min/L)	SD
PAN PEI	43,627.02	1532.896	PAN PEI	42,933.61	5979.0174
PS	54,475.07	2423.964	PS	47,929.42	5048.3368
PAN	55,784.54	1020.386	PAN	45,734.66	9467.0715

**Table 5:** Differences in final and initial linezolid concentrations in 0.9% NaCl samples—percentage value.

Membrane Type	Concentration T0	Concentration T120	Difference T120/T0 (%)
PS	637.43	507.43	80%
PAN	542.54	370.18	68%
PAN PEI	594.74	310.66	52%

**Table 6:** Statistics of one way ANOVA + Tukey’s post hoc test—bovine blood AUC.

Group	Significance	*p* Value
PAN PEI vs. PS	YES	*p* < 0.001
PAN PEI vs. PAN	YES	*p* < 0.001
PS vs. PAN	NO	*p* = 0.653

**Table 7:** Statistics for one way ANOVA + Tukey’s post hoc test—0.9% Natrium Chloratum AUC.

Group	Significance	*p* Value
PAN PEI vs. PS	NO	
PAN PEI vs. PAN	NO	
PS vs. PAN	NO	

**Table 8:** Extraction ratio.

50 ng/mL				50,000 ng/mL			
Xi	X	100-(Xi/X × 100%)	Absolute value	Xi	X	100-(Xi/X × 100%)	Absolute Value
575.794	547.91	−5089	5089	60,371.86	53,446.117	−12,958	12,958
587.827	545.974	−7666	7666	61,340.94	54,516.504	−12,518	12,518
555.931	551.025	−0.89	0890	62,688.19	52,731.098	−18,883	18,883
569.699	539.78	−5543	5543	62,502.66	53,562.961	−16,690	16,690
598.727	504.832	−18,599	18,599	64,900.18	56,983.543	−13,893	13,893
617.347	580.592	−6331	6331	63,411.58	56,510.918	−12,211	12,211
	Mean		7353		Mean		14,526

Xi—area under the curve for samples prepared on plasma, where the analyte was added after extraction. X—area under the curve for samples prepared in water, where the analyte was added after extraction.

**Table 9:** Total recovery.

50 ng/mL			50,000 ng/mL		
Xz	Xi	Recovery	Xz	Xi	Recovery
634,239	575,794	110,150	620,620.477	703,718.563	88,192
592,218	687,827	86,100	638,306.074	713,409.438	89,473
584,745	555,931	105,183	599,047.836	656,881.938	91,196
618,035	569,699	108,484	595,142.809	665,026.563	89,492
616,452	598,727	102,960	590,911.598	649,001.813	91,049
547,884	617,347	88,748	601,217.789	674,115.813	89,186
	Mean	100,271		Mean	89,764
	SD stand	10,296		SD stand	1155

Xz—area under the curve for the plasma sample to which analytes were added before extraction. Xi—area under the curve for the plasma sample to which the analytes were added after extraction.

## Data Availability

The data presented in this study are available on request from the corresponding author.
